# Colonic Intramural Hematoma in a Cat: A Case Report

**DOI:** 10.3389/fvets.2022.913862

**Published:** 2022-06-17

**Authors:** Ti-Chiu Hsu, Lee-Shuan Lin, Cheng-Shu Chung, Chuan Chiang, Hsien-Chieh Chiu, Ping-Hsun Huang

**Affiliations:** ^1^Laboratory of Veterinary Diagnostic Imaging, Department of Veterinary Medicine, College of Veterinary Medicine, National Pingtung University of Science and Technology, Pingtung, Taiwan; ^2^Division of Diagnostic Imaging, Veterinary Medical Teaching Hospital, National Pingtung University of Science and Technology, Pingtung, Taiwan; ^3^School of Dentistry, College of Dental Medicine, Kaohsiung Medical University, Kaohsiung, Taiwan; ^4^Department of Medical Imaging and Radiological Sciences, College of Health Sciences, Kaohsiung Medical University, Kaohsiung, Taiwan; ^5^Laboratory of Veterinary Surgery, Department of Veterinary Medicine, College of Veterinary Medicine, National Pingtung University of Science and Technology, Pingtung, Taiwan; ^6^Division of Small Animal Surgery, Veterinary Medical Teaching Hospital, National Pingtung University of Science and Technology, Pingtung, Taiwan; ^7^UniCore Animal Hospital, Taipei City, Taiwan; ^8^Tzuoo Ann Animal Hospital, Taipei City, Taiwan

**Keywords:** cat, colonic intramural hematoma, colonoscopy, computed tomography, ultrasonography

## Abstract

Colonic intramural hematoma is a rare condition in humans and companion animals. Its clinical presentation in cats has not previously been reported. An 8-year-old male American shorthair cat presented with acute onset of constipation and anorexia for 3 days. Laboratory examination indicated mild elevation of alanine aminotransferase, globulin, and total protein levels. Complete blood count was normal. Radiographs revealed a soft tissue opacity mass located caudodorsally to the urinary bladder, causing narrowing of the descending colonic lumen. Sonography showed a heteroechogenic intraluminal mass containing liquefied content between the submucosal and muscular layers of the descending colon. On computed tomographic images, the mass contained two different attenuated contents with an interface. Colonoscopy was then performed for intestinal biopsy, and the contents observed in the intraluminal mass were drained *via* surgical evacuation and considered as blood clots. Supportive medical treatment, including antibiotics and fecal softener, was administered, and the clinical signs resolved uneventfully. Mild chronic proctitis without apparent malignancy was confirmed histopathologically, and no recurrence was observed after more than 14 months, and thus a colonic intramural hematoma was presumptively diagnosed. The information provided by multimodal imaging of the mass was essential for the diagnosis and determination of the treatment in this case.

## Introduction

Colonic intramural hematoma is rare in humans and animals and occurs mostly secondary to blunt abdominal trauma, anticoagulant therapy, coagulopathies, or intestinal neoplasia in humans (1). In humans, spontaneous colonic hematoma is most commonly associated with blunt trauma (2). Risk factors include bleeding diathesis, such as those experienced by patients undergoing anticoagulant therapy or with coagulopathies (3). Few cases secondary to synchronous colon cancer for patients who underwent hemicolectomy have also been reported (1, 4). Rarely, it occurs secondary to vaginal delivery (5). Clinically, colonic intramural hematoma usually causes lower abdominal pain, lethargy, anorexia, and constipation in humans. The diagnosis of colonic intramural hematoma in humans is mainly based on radiology, such as contrast-enhanced computed tomography (CT) (1). Treatment may vary from case to case, including surgical and conservative management; however, its clinical presentation, diagnosis, and treatment outcome have not been previously described in cats. Therefore, this report aimed to present the multimodal imaging findings of colonic intramural hematoma in a cat and to describe the successful treatment with surgical evacuation.

## Case Description

An 8-year-old neutered male American shorthair cat was referred to the Unicore Animal Hospital because of decreased defecation for 1 week and acute onset of constipation with anorexia 3 days prior. Physical examination revealed no apparent colonic narrowing on rectal palpation.

Laboratory examination was performed for preanesthetic evaluation and revealed mild elevated levels of alanine aminotransferase (152 U/L; reference range, 12–130 U/L), globulin (5.9 g/dl; reference range, 2.8–5.1 g/dl), and total protein (9.3 g/dl; reference range, 5.7–8.9 g/dl). The complete blood count was normal.

**Figure 1:**
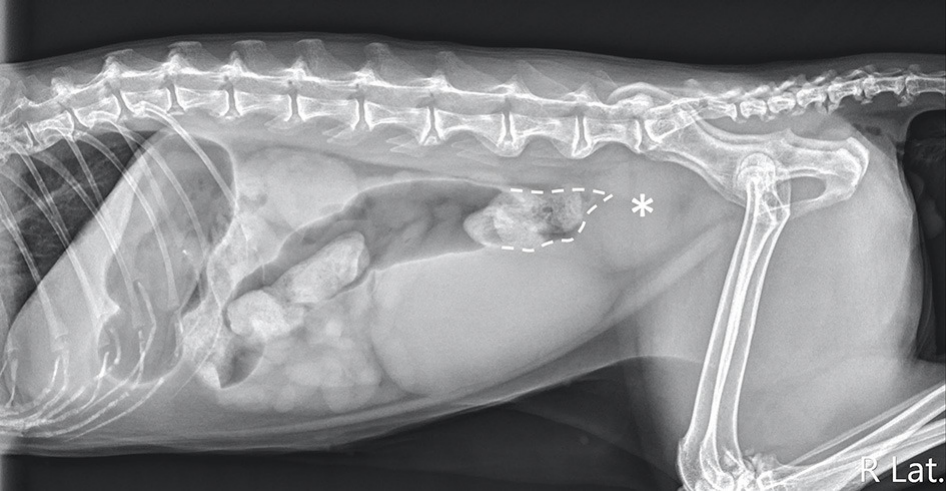
Right lateral projection of the abdomen. An ovoid-shaped, soft-tissue opacity mass (asterisk) caudodorsal to the urinary bladder was noted, causing a narrowing of the descending colonic lumen (dotted line).

Abdominal radiography (MODEL BLR-500A; Toshiba, Tochigi, Japan) of the right lateral, left lateral, and ventrodorsal projections was performed while the cat was awake, and a large, well-defined, ovoid-shaped, soft tissue opacity mass was observed caudodorsal to the urinary bladder at the sixth lumbar vertebra to the sacrum level, causing dorsal displacement, and narrowing of the descending colon (Figure 1). The mass was approximately 3.1 cm in height and 3.6 cm in length in the right lateral abdominal projection. Dilation of the colon was observed, with gas accumulation cranial to the narrowed location. Radiographic differential diagnoses included a colonic intraluminal neoplasia/foreign body, an intramural neoplasia/abscess/hematoma, or an extramural neoplasia/granuloma. Considering the rapid progression of the disease, abscesses and hematomas were mostly suspected.

**Figure 2:**
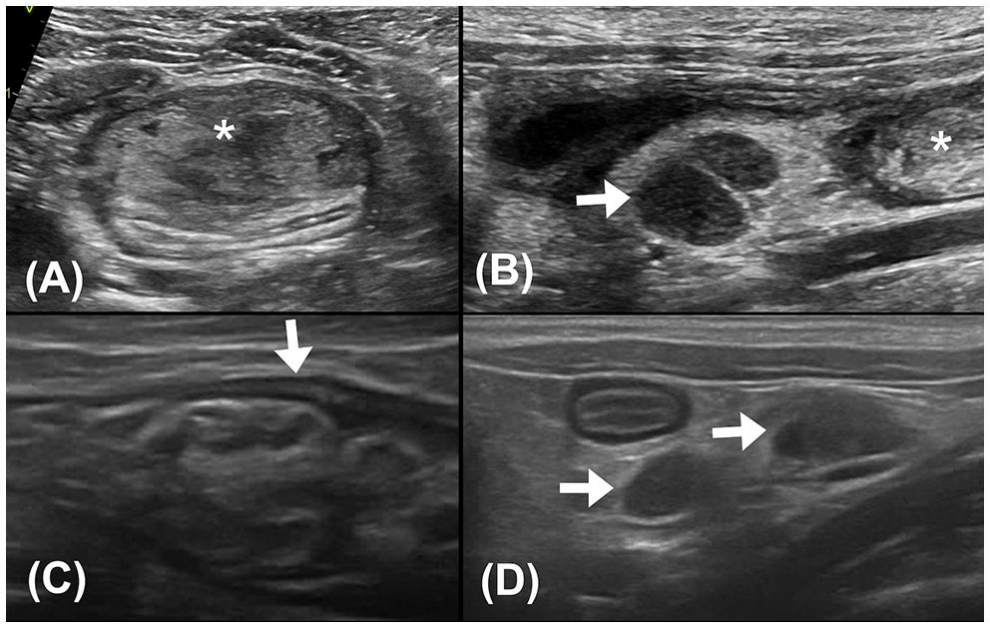
**(A)** Transverse plane ultrasound image of the descending colon. A hyperechoic mass (asterisk) with heteroechogenicity between the submucosal and muscular layers of the descending colon was seen. **(B)** Sagittal plane ultrasound image of the colonic lymph nodes (0.6 cm in thickness and 0.9 cm in length). The colonic lymph nodes (arrow) adjacent to the mass (asterisk) appeared enlarged and hypoechogenic. **(C)** Follow-up sagittal plane ultrasound image of the descending colon 2 weeks after surgical evacuation. No mass effect was noted at the affected site. The intestinal layering (arrow) was relatively normal, with a thickened and corrugated mucosal layer. **(D)** Follow-up sagittal plane ultrasound image of colonic lymph nodes 2 weeks after surgical evacuation. The colonic lymph nodes (arrows) were smaller (0.4 cm in thickness and 0.7 cm in length) and with homogeneous echogenicity.

Abdominal ultrasonography was performed with an ultrasound device (Vivid E80; GE Healthcare, IL, USA) equipped with a linear transducer (11 L-D, GE Healthcare; 4–10 MHz), and the patient was positioned in dorsal recumbency under general anesthesia. The sagittal and transverse planes of ultrasonographic images revealed a focal heteroechogenic intramural mass located between the submucosal and muscular layers of the descending colon (~2.6 cm in width and 1.3 cm in height), causing extreme compression and occlusion of the colonic lumen. The intramural mass was liquefied and floating. Enlarged colonic lymph nodes were observed cranial to the intramural colonic mass, indicating hypoechogenicity (0.6 cm in thickness and 0.9 cm in length). Considering the location and echogenicity of the mass, colonic intramural neoplasia with regional colonic lymphadenopathy was mainly suspected, whereas abscess and hematoma could not be completely excluded (Figures 2A,B).

**Figure 3:**
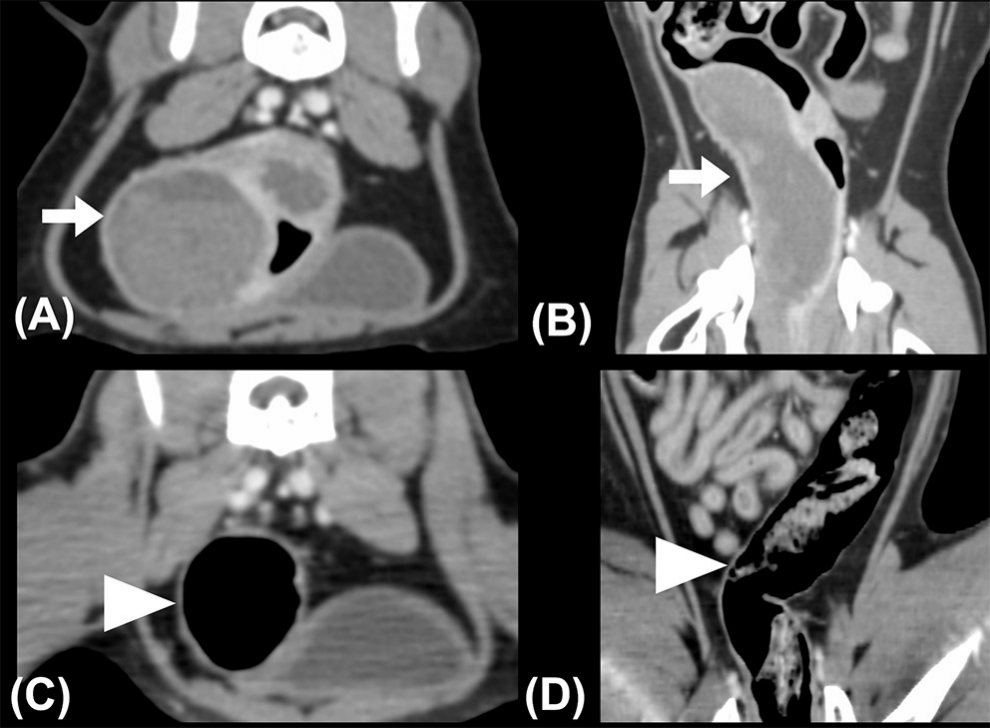
**(A)** Transverse and **(B)** dorsal plane CT images revealed intramural masses (arrows) in the descending colon. Hyperattenuating contents accumulated on the gravity-dependent side of the mass, exhibiting two different layers within the mass with a clear interface. The whole descending colon was deviated to the right on the pre-treatment CT images. **(C)**Transverse and **(D)** dorsal plane CT images obtained 1-month post-treatment. Normal colonic intestinal layering was noted, while the colonic lymph nodes (not shown) appeared normal in size and attenuation. The descending colon was located in the left caudal abdominal cavity. The patient's right side is shown on the left image.

A triple-phase CT examination (Discovery 690; GE Healthcare, IL, USA) was performed to obtain further information. On CT images, a large, fluid-to-soft tissue attenuating mass was noted between the submucosal and serosal layers, arising from the middle portion of the descending colon, extending caudally to the distal descending colon immediately cranial to the rectum, and spanning approximately 8 cm in length. The colonic intramural mass contained two different attenuating contents with an interface at all three phases: hyperattenuating materials (40–60 Hounsfield Unit, HU) accumulating on the gravity-dependent side and lower attenuating contents (30–40 HU) on the upper side (Figures 3A,B). Ultrasound-guided fine-needle aspiration was performed, and ~8 ml of non-agglutinated blood was drained. The tentative diagnosis was intraluminal hematoma based on cytologic results.

**Figure 4:**
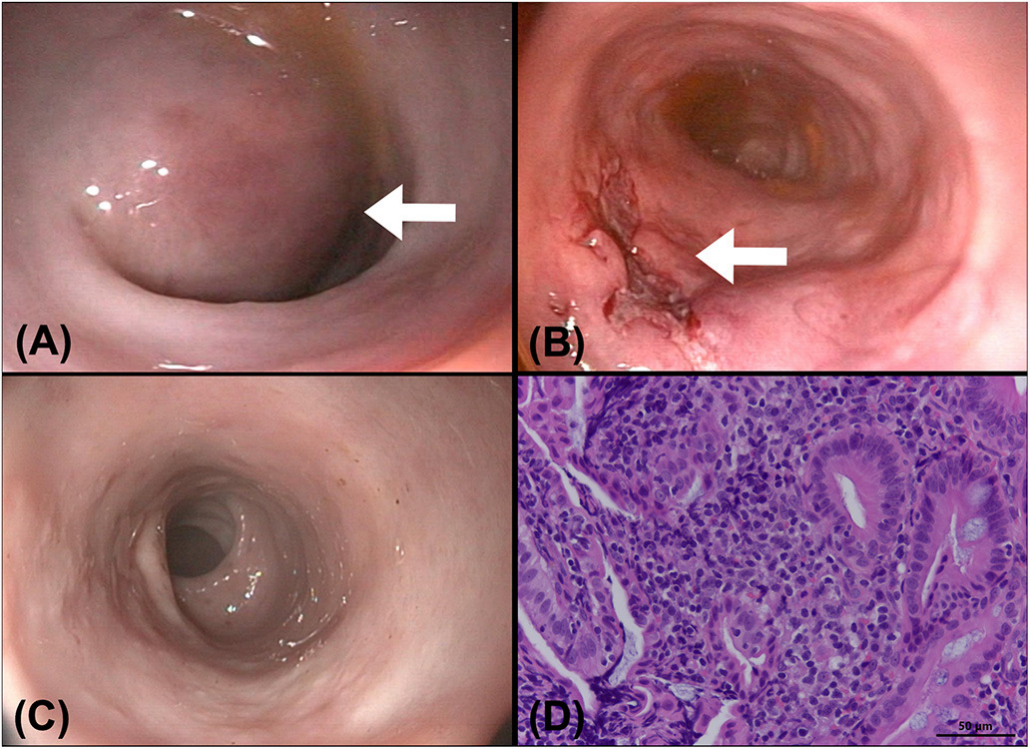
A colonoscopy was performed after a CT examination. **(A)** A round submucosal mass (arrow) with an intact mucosa was noticed at the descending colon, causing narrowing of the colonic lumen. **(B)** The colonoscopic image obtained after surgical evacuation of the contents in the mass: the incision wound (arrow) was kept open for drainage of the remaining blood clots. **(C)** Image of a follow-up colonoscopy 1 month after evacuation: the incision wound was well-healed, and no recurrence was seen. **(D)** Pathology of the colonic specimen showed chronic proctitis and lymphoid hyperplasia of the colon. A small number of lymphocytes and plasma cells with fewer neutrophils are noted multifocally in the lamina propria of colon (H&E stain, bar = 50 μm, 400×).

Lactulose (2 ml per time, SID, PO) was prescribed for 1 week to resolve constipation. The cat underwent colonoscopy (EVIS EXERA III, CLV-190, PCF-PH190L, Olympus Corporation, Tokyo, Japan) 1 week later at the Tzuoo Ann Animal Hospital, and a mass with an intact mucosal surface and spotty oozing blood was observed (Figure 4A). A speculum was used to approach the mass from the anus and make an incision with a scalpel because it was difficult to penetrate the mass with biopsy forceps. A large number of blood clots were aspirated from the mass. A cotton swab was used to confirm the integrity of the outer intestinal wall and intestinal biopsies of the lesions were sampled with Blakesley nasal forceps (N2990, STORZ, Tuttlingen, Germany). Colonoscopy was reperformed to ensure that there was no active bleeding after surgical evacuation and biopsy (Figure 4B). Histologically, the specimens revealed that mucosal architectures and multiple hyperplastic lymphoid foci were noted at the junction of the mucosa and submucosa. A small number of multifocal lymphocytes and plasma cells were also noted in the lamina propria, with fewer neutrophils. The patient was diagnosed as chronic proctitis with lymphoid hyperplasia (Figure 4D).

**Figure 5:**
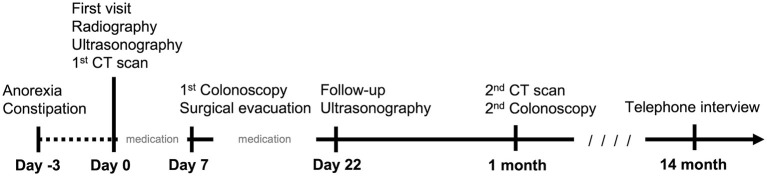
A brief timeline of the medicine intervention in the cat. Clinical signs presented 3 days prior to first visit. Radiography, ultrasonography, and CT examination were performed on the same day of the first visit. Palliative medication was administrated for 7 days. The surgical intervention was performed 7 days later. Post-operative medicine was administrated for 2 weeks. The patient revisited on day 22. A follow-up CT scan and colonoscopy were conducted 1 month later. After 14 months, a final telephone interview was conducted.

After colonoscopy, amoxicillin (20 mg/kg, BID, PO), lysozyme (17 mg/kg, BID, PO), L-glutamine (27 mg/kg, BID, PO), tranexamic acid (20 mg/kg, BID, PO), and lactulose (2 ml per time, SID, PO) were prescribed for 2 weeks. Constipation resolved after surgical evacuation, only scant blood was observed in the stool for 4 days. No apparent defecation difficulty, anorexia, or abdominal pain was noted. Follow-up ultrasonography (MyLab^TM^ Class C, LA435, 6–18 MHz, Esaote, Italy) performed 2 weeks later at the Tzuoo Ann Animal Hospital revealed that the affected colon appeared to display normal layering with thickened and corrugated mucosa. The colonic lymph nodes showed homogeneous echogenicity and smaller size than before (0.4 cm in thickness and 0.7 cm in length; Figures 2C,D). A follow-up CT (Aquilion, Toshiba Medical Systems Corporation, Tochigi, Japan) (Figures 3C,D), colonoscopy (Figure 4C), and laboratory examination were performed 1 month later, and showed no mass effect or abnormality during the examinations, while globulin content returned to normal levels and blood coagulation was normal. No relative clinical signs were noted in follow-up telephone interviews after 14 months. The timeline of medical interventions is illustrated in Figure 5.

## Discussion

Intestinal intramural hematoma is rare in companion animals, and only five cases have been reported in dogs in three previous studies (6–8). Based on our knowledge, this is the first report describing intestinal intramural hematoma in cats. Intestinal intramural hematomas may occur in any part of the intestine. Small intestinal intramural hematomas in dogs are mostly associated with pancreatitis or anticoagulant therapy, resulting in upper abdominal pain and vomiting. In the literature on human patients, the small intestine is affected much more frequently than the large intestine, due to the fixed position of the duodenum anterior to the vertebral column, the force of a blow tends to shear the duodenum held in this fixed position in traumatic cases (6, 9). Additionally, pancreatic disease has been associated with intramural hematoma of the duodenum (9, 10). Colonic intramural hematomas are mostly caused by blunt abdominal trauma, anticoagulant therapy, coagulopathies, or intestinal neoplasia, and large colonic hematomas lead to colonic obstruction, resulting in lower abdominal pain, decreased defecation, vomiting, nausea, and abdominal distention (1). No colonic intramural hematoma has been reported in dogs or cats. In our case, the hematoma was located in the descending colon, causing obstruction of the colonic lumen and resulting in a progressively decrease in defecation and acute onset of constipation with anorexia.

Anticoagulant therapy, foreign bodies, coagulopathies, and secondary blunt abdominal trauma are common causes of colonic hematoma in humans (11). Hematological examination showed a normal coagulopathy function and no clinical history of associated anticoagulation therapy in our case, and no specific findings regarding foreign bodies were noted in the whole digestive system. However, blunt abdominal trauma could not be completely excluded, although it might not have been noticed by the owner. Colonic intramural hematoma induced by blunt abdominal trauma appeared with no apparent cutaneous wound on the first day in human patients, but the acute onset of lower abdominal pain and constipation occurred 1 day later, which is similar to the acute onset of constipation in our case (1, 11). In the veterinary literature for dogs and horses, small intestinal intramural hematomas are usually noted within the tunica muscularis or between the submucosa and tunica muscularis; whereas large intestinal intramural hematomas in houses are usually located in the submucosa (6, 8, 12–14). The intestinal mucosa contains numerous blood and lymph vessels, and hematomas are produced at the tunica muscularis and submucosal layer upon tearing of the terminal artery vessels as they leave the mesentery to penetrate the muscular layer of the intestinal wall, which may explain why the colonic intramural hematoma in this cat was located between the submucosa and muscular layer on ultrasonographic images (15–18). However, in our case, only the mucosa and submucosa could be pathologically evaluated because an invasive procedure for full-thickness intestinal biopsy was not performed at the owner's request.

In our case, a large soft tissue opacity mass was noted in the caudal abdomen, causing dorsal deviation and extreme occlusion of the descending colon and leading to megacolon, which is mostly associated with constipation. Considering the location, the mass was mainly suspected to have arisen from the distal descending colon. However, radiographs only show the location of the mass and cannot differentiate whether it is intraluminal, intramural, or extramural mass. Considering the acute onset of clinical signs, hematoma, abscess, and foreign body were suspected and the likelihood of a granuloma is lower (19). Neoplasia could not be completely excluded, due to the variable duration of onset of clinical signs (20–22). Further diagnostic imaging examinations are required to identify the origin of the mass.

This cat showed a heterogeneous echoic, liquefied mass between the submucosal and muscular layers in the descending colon with regional colonic lymphadenopathy. The echogenicity was similar to that observed in feline colonic neoplasia or feline gastrointestinal eosinophilic sclerosing fibroplasia (FGESF); however, inconsistent with the loss of layering, our case showed intact intestinal layering (23–25). Fluid-filled contents may indicate an abscess; however, homogeneous or anechoic contents are more commonly seen in abscesses (24, 26, 27). In our case, radiographic and ultrasonographic findings together confirmed a colonic intramural mass between the submucosal and muscular layers, but differentiation from the hematoma sonographically was difficult, as similar lesions might also present in intramural neoplasia, abscesses, and FGESF. Considering the large extent of the mass and multiple colonic lymphadenopathies, a more comprehensive examination, such as CT, was performed to provide more information.

On CT images, a large, well-capsuled, soft-tissue attenuating mass was noted in the submucosal and serosal layers of the distal descending colon, causing severe narrowing of the colonic lumen. The mass was nonenhancing and showed stratified contents with a clear interface, 30–40 and 40–60 HU in the upper and lower layers, respectively, similar to that seen in the human literature (28). This interface was only seen on CT images but not on ultrasonography, which might be caused by precipitation of lysed and fragmented clots upon ventral recumbency of the patient during CT scans. Cytological examination revealed abundant red blood cell contents without platelets, consistent with previous chronic hemorrhage (29). A clear interface with different attenuations was also observed in large intracranial hematomas in humans, although the lesion locations were different (30). In our case, triple-phase contrast was performed, and the mass showed no enhancement and intact mucosal and serosal intestinal layers in the arterial, portal, and delayed phases, which was similar to the characteristics of the human colonic intramural hematoma on CT images and further excluded the suspicion of intestinal neoplasia and FGESF (11, 31–33). Abscesses were also excluded due to higher attenuation of contents in the mass (24). The CT image in our case provided a more complete visualization of the abdomen than ultrasonography and radiography and was helpful in excluding intestinal perforation and active bleeding to determine whether emergency surgery was necessary.

The relationship between the timing of hematoma and image characteristics is well known in the human literature; however, it is less well described in animals. In humans, hematomas present variable echogenicity and density at different times on ultrasonographic and CT images (31, 34). Immediately after the hemorrhage (on day 1), freshly extravasated blood is mostly anechoic on ultrasonography and exhibits mixed 40–80 HU signals on CT images, which can be attributed to the formation of a meshwork of fibrin fibrils and globin molecules (3, 31). During week 1, as the hematoma matures, clot retraction ensues, the hematoma shows mostly heterogeneous echogenicity on ultrasonographic images and shows 50–80 HU signals on the CT image, which is similar to what was seen in our case (31, 34). The hematoma gradually becomes more hypoechogenic on ultrasonographic images and hypoattenuating (~10–50 HU) on CT images after the acute phase due to chemical breakdown of globin molecules and lysis of clots over the weeks (31, 34). The intramural mass in our case showed heterogeneous echogenicity on ultrasonography and intermediate attenuation on CT images; therefore, we speculated that the hematoma was formed within 1 week, and this time period also coincided with the onset of clinical signs.

In humans, the treatment of intramural colonic hematoma includes conservative and surgical treatment, whereas descriptions in animals are limited. The optimal management of colonic intramural hematoma depends on the differences in etiologies and the patient's general condition in humans. Conservative treatment is typically used, especially when coagulopathy is the cause (1, 3). Surgical intervention includes surgical excision and surgical evacuation. Surgical excision, such as in hemicolectomy, is often required in cases showing failure of conservative treatment, peritonitis, hemoperitoneum, and intractable bleeding; whereas surgical evacuation may be considered in cases without mucosal perforation (18). Evacuation solely by colonoscopy is rarely seen in the literature because it is difficult to detect and sample colonic intramural hematoma in deeper layers, such as the muscular layer (6, 35). Surgical evacuation of the hematoma by laparotomy is more feasible, because it can facilitate a direct approach of the lesion and ensure there is no oozing after evacuation. Complications of surgical evacuation include hemoperitoneum, peritonitis, and intestinal perforation (18). In our case, ultrasonography revealed the location of the mass was between the submucosal and muscular layers, and the thickness of the mass was more than 1.3 cm, thus we considered incision from the mucosal surface for surgical evacuation was safe. The distal location of the hematoma enabled the anal approach; therefore, surgical evacuation from the anus with a speculum was performed. A cotton swab was used to ensure that the outer colonic layers were not injured or perforated. The wound was not closed for the drainage of the remaining blood clots. After surgery, antibiotics and hemostatic medicine were administered, and the clinical signs, including appetite, spirits, defecation, and abdominal pain, were followed up to ensure that there were no postsurgical complications. No recurrence or complication was noted for more than 14 months; therefore, we considered that this was a sporadic disease in this cat, and surgical evacuation was feasible in this situation.

There are several limitations to our case report. First, no full-thickness biopsy was performed in our case to confirm the origin of the hematoma. Second, the cause of hematoma in this cat is still unknown, thus the incidence of recurrence cannot be estimated. Last, although the outcome of surgical evacuation was good in the current case, as an individual case study, the contraindication of surgical evacuation cannot be fully confirmed.

## Conclusion

In conclusion, our report describes the multimodality imaging findings and their clinical relevance in a rare case of feline colonic intramural hematoma. The mass appeared as a large mass affecting the descending colon on radiographs, an intraluminal mass between the submucosal and muscular layers with heteroechogenicity and liquified contents on ultrasonography, and showing almost non-enhancing and stratified contents on CT images. Ultrasonography provides a more precise layering location, whereas enhancement and density in CT images can offer more definitive information regarding the diagnosis and formation of the hematoma. The patient responded well to surgical evacuation of the hematoma using the anal approach. No recurrence was noted for more than 14 months. The multimodal imaging findings in this case were invaluable for differential diagnosis and determining the optimal course of treatment.

## Data Availability Statement

The original contributions presented in the study are included in the article/Supplementary Material, further inquiries can be directed to the corresponding author.

## Ethics Statement

Ethical review and approval were not required for the animal study because this was a retrospective case report. Written informed consent was obtained from the owners for the participation of their animals in this study.

## Author Contributions

T-CH, L-SL, CC, H-CC, and P-HH contributed to image acquisition. T-CH, L-SL, and CC contributed to image interpretation. H-CC and P-HH performed the colonoscopy and surgical intervention. T-CH, L-SL, and C-SC contributed to manuscript editing. All authors reviewed and approved the final submitted manuscript.

## Funding

This publication was supported by the Southern Taiwan Science Park Bureau, Ministry of Science and Technology, Taiwan, ROC under contract 110CB-1-04.

## Conflict of Interest

The authors declare that the research was conducted in the absence of any commercial or financial relationships that could be construed as a potential conflict of interest.

## Publisher's Note

All claims expressed in this article are solely those of the authors and do not necessarily represent those of their affiliated organizations, or those of the publisher, the editors and the reviewers. Any product that may be evaluated in this article, or claim that may be made by its manufacturer, is not guaranteed or endorsed by the publisher.

## Supplementary Material

Supplementary Video I Ultrasonographic images of the colonic intramural hematoma and colonic lymphadenopathy.
